# Psychometric Qualities of the McMaster Family Assessment Device–General Functioning Subscale for Malaysian Samples

**DOI:** 10.3390/ijerph19042440

**Published:** 2022-02-20

**Authors:** Chin Wen Cong, Soon Aun Tan, Sarvarubini Nainee, Chee-Seng Tan

**Affiliations:** Department of Psychology and Counselling, Faculty of Arts and Social Science, Universiti Tunku Abdul Rahman (UTAR), Kampar 31900, Perak, Malaysia; sarvarubini@utar.edu.my (S.N.); cstan@outlook.my (C.-S.T.)

**Keywords:** family assessment device, family functioning, general functioning, Malaysia, psychometrics

## Abstract

Family functioning has been associated with psychological well-being and physical health. The 12-item McMaster Family Assessment Device–General Functioning Subscale (FAD-GF) has been widely used to assess individuals’ overall level of family functioning. However, it has shown an inconsistent factor structure across various studies. The present study investigated its psychometric qualities in two studies with two different adult samples in Malaysia. In Study 1 (N = 417, 55.3% females, 19 to 26 years old), exploratory factor analyses were conducted, and four models were found: a three-factor model with 11 items, a two-factor model with 12 items, and one-factor models with six negatively worded items and six positively worded items, respectively. Study 2 (N = 358, 65.1% females, 18 to 60 years old) compared models found in past studies and those found in Study 1 through confirmatory factor analyses on another sample of adults. Among the six competing models, the two-factor model with three positively worded and three negatively worded items (i.e., FAD-GF-SF) is preferable because it did not require modification and showed a clear-cut result of goodness of fit. The subscales demonstrated satisfactory internal consistency. In conclusion, the FAD-GF-SF is a useful instrument for measuring family functioning in the Malaysian context.

## 1. Introduction

Family is important in setting the primary environment to productively nurture an individual for society. The healthiness of a family unit can be recognized through the unit’s ability to treat problems together and to include each member’s responsibilities towards the rest of the unit in its routine [1]. Family functioning is thus viewed as a unit with an intense connection that covers the cohesiveness, closeness, interactions, and relationship within members in a family environment [2]. On a simpler note, family functioning refers to how families cope with their daily routines by fulfilling their roles in their family [3]. Several studies have found family functioning has a positive relationship with well-being [4], resilience [5], and physical activity [6] besides a negative relationship with loneliness [7], depression [8], Internet addiction [9], child body mass index [10], and suicidal ideation [11].

High family functioning is associated with high resilience practice. Generally, building resilience is a dynamic process whereby having a good relational bases (i.e., family functioning) could provide encouragement and reassurance for an individual at instant which in turn may build one’s resiliency [12]. Given its important role in individuals’ psychological well-being and physical health, some measurements have been developed for researchers to investigate family functioning, for example, Family Environment Scale [13], Family APGAR [14], and Family Functioning Index [15]. Among the existing measurements, the McMaster Family Assessment Device (FAD) [16] has been widely used across various populations (e.g., nonclinical, psychiatric, and medical samples) and cultural groups [17,18,19]. To our best knowledge, the FAD is the only measurement that has a subscale that specifically assesses the general family functioning (i.e., General Functioning Subscale).

### 1.1. McMaster Family Assessment Device–General Functioning Subscale (FAD-GF)

The McMaster Family Assessment Device (FAD) [16] was developed based on the McMaster Model of Family Functioning (MMFF) [20]. The FAD consists of seven subscales: (1) Problem-Solving (i.e., household member’s ability to resolve problems for a good family functioning), (2) Communication (i.e., information exchange among household members), (3) Roles (i.e., whether the family has recurrent patterns of behavior to handle family functions), (4) Affective Responsiveness (i.e., the household member’s ability to react with proper emotion to environmental stimuli), (5) Affective Involvement (i.e., the extent of warmth among family members), (6) Behavioral Control (i.e., whether the family has norms or standards leading individual responses to emergencies), and (7) General Functioning (i.e., the overall level of family functioning).

Among the seven subscales, the General Functioning Subscale (FAD-GF) has been frequently administered to Malaysian samples (e.g., [21,22,23]). The 12-item FAD-GF is a unidimensional measure that evaluates the general pathology of a family with six items on healthy and unhealthy family functioning, respectively [16]. Thus far, it has been translated into different languages such as Spanish [24], Italian [25], French [26], Chinese [27], and Bahasa Malaysia [21]. The FAD-GF has been found to have good internal consistency [16,23].

Nevertheless, different factor structures of the FAD-GF have been found, ranging from the original 12-item one-factor model [16,23], a one-factor model with six positively worded items [28], a two-factor model with six items (three positively and negatively worded items, respectively) [21], to four-factor models (i.e., competence in family functioning, emotional communication, centered-on-self behavior, and emotional and behavior over-involvement) with 12 items [25]. Since a reliable and valid measurement is essential in extending our understanding of family functioning and its beneficial role, it is imperative to identify the factor structure and examine the psychometric qualities of the FAD-GF that works in the local context for Malaysian samples. This in turn could provide a cross-cultural re-validation of the scale.

### 1.2. Overview of the Present Study

The present study aimed to examine and identify the factor structure of the FAD-GF for Malaysian adults in two studies. First, we investigated the factor structure of the FAD-GF using exploratory factor analysis (EFA) in Study 1. Then, in Study 2, we examined and compared competing models, including those derived from Study 1 using confirmatory factor analysis (CFA) to identify the best-fit model. Besides that, the internal consistency, structural validity, and concurrent validity of the retained model were also tested.

## 2. Study 1: Exploratory Factor Analysis

Psychometric studies on the FAD-GF have revealed different versions of the factor structure. Therefore, we began with EFA to explore potential models that enable us to include the competing models and to have a comprehensive evaluation in Study 2.

### 2.1. Participants and Procedure

An online survey was used and a total of 478 adults participated in this study. However, 61 participants submitted empty surveys and thus were removed. The final sample consisted of 417 Malaysian adults (55.3% females, one participant did not report gender). The average age of the samples was 21.06 (*SD* = 1.56), ranging from 19 to 26 (one participant did not report his or her age). Among them, 104 (25%) identified themselves as Malays, 182 (43.7%) as Chinese, 121 (29.1%) as Indians, 9 (2.2%) as others (e.g., Bidayuh, Kadazan, Punjabi), and one missing value. The majority of the participants (78.3%) were single and the rest reported that they are currently in a relationship (21.7%). Six participants did not report their relationship status. The participants were recruited using convenience sampling. Participants were given a standard information sheet and informed consent before answering the questions. The data collection procedure was reviewed and approved by the Institutional Scientific and Ethical Review Committee.

### 2.2. Measurements

The McMaster Family Assessment Device–General Functioning Subscale (FAD-GF) [16] was employed in Study 1. Respondents were required to answer 12 items using a 4-point Likert scale ranging from 1 (strongly disagree) to 4 (strongly agree). Sample items are “We cannot talk to each other about sadness we feel” (negative item), and “We are able to make decisions about how to solve problems” (positive item). A mean score is computed by averaging the item scores after reverse scoring six negatively worded items. Higher scores indicate a higher level of healthy family functioning.

### 2.3. Data Analysis

The responses were submitted to exploratory factor analysis (EFA) using SPSS version 25 to examine the factor structure underlying the 12-item FAD-GF.

### 2.4. Results

Inspection of the dataset discovered four items with missing values. As the percentage of missing value was low (i.e., 0.2% for each item) and the Little’s MCAR test was not statistically significant: χ^2^ (4) = 8.86, *p* = 0.065, we replaced the missing data using expectation maximization (EM).

A series of EFA was conducted to examine the factor structure of the 12-item FAD-GF. The first EFA was conducted using principal axis factoring (PAF), Promax rotation (which allows factors to be correlated), and the Kaiser–Guttman rule (i.e., eigenvalue > 1.0). The Kaiser–Meyer–Olkin (KMO) value was 0.810, exceeding the recommended value of 0.60 [29]. The Bartlett’s test was also statistically significant, χ^2^ (66) = 1089.33, *p* < 0.001, thus supporting the appropriateness of conducting factor analysis on the items. Three factors were extracted, and they explained 53.72% of the total variance. All the factor loadings were greater than 0.40 except for item 12 (“We confide in each other”). Following the cut-off point of 0.40 [30,31], item 12 was then removed and EFA with the remaining 11 items was undertaken. Again, a three-factor model was found and explained 56.62% of the total variance. All the 11 items showed a clear loading on one of the three factors (see Table 1). The first factor (negative functioning) with items 1, 7, 9 and 11 accounted for 30.68% of the variance (eigenvalue = 3.375), whereas the second factor (positive functioning) with items 2, 4, 8 and 10 explained 16.09% of the variance (eigenvalue = 1.770). The remaining three items (items 3, 5 and 6) loaded on the third factor (emotional communication) and explained 9.85% of the total variance (eigenvalue = 1.084).

Although the results of the three-factor model were satisfactory, the eigenvalue is not without limitation (e.g., [32]). Therefore, we conducted another EFA using PAF and Promax rotation to examine a two-factor model suggested by parallel test [33]. Factor loadings of the 12 items were greater than 0.40 and the items loaded clearly on one of the two factors. The two-factor model explained 44.50% of the total variance. The first factor (negative functioning) comprised all the negatively worded items and explained 28.63% of the total variance (eigenvalue = 3.436). On the other hand, the second factor (positive functioning) which consisted of all the positively worded items explained 15.87% of the total variance (eigenvalue = 1.904).

The third EFA was then performed using PAF and Varimax rotation by fixing the number of extractions to examine if the hypothetical one-factor model (with 12 items) could be found. The one-factor model explained 28.64% of the total variance. We removed items with factor loadings below 0.40 and reran EFA with the remaining items until all items achieved satisfactory factor loadings. Consequently, all positively worded items were removed. The one-factor model with six negatively worded items explained 48.19% of the total variance (eigenvalue = 2.891).

Finally, we explored another one-factor model with the six positively worded items (items 2, 4, 6, 8, 10, 12) revealed by Haan et al. [28] using PAF and Varimax rotation. All items showed factor loadings greater than 0.40. The model explained 39.96% of the total variance (eigenvalue = 2.398). Table 1 summarizes the results of the EFAs.

The FAD-GF is supposed to be a unidimensional (sub-)scale with 12 items. However, EFAs based on eigenvalues and O’Connor’s [33] parallel test revealed a three-factor model with 11 items and a two-factor model with 12 items, respectively. Furthermore, two one-factor models were also found with the respective negatively worded items and positively worded items. Such inconclusive results imply that some FAD-GF items may not be applicable in the Malaysian context and highlight the need for further examination to verify the factor structure of the FAD-GF for the Malaysian sample.

## 3. Study 2: Confirmatory Factor Analysis

Four alternative models, but not the hypothetical 12-item one-factor model, were displayed in Study 1. Therefore, Study 2 aimed to identify the best fit model on a new sample by comparing six possible models including the four models found in Study 1, the two-factor model with three positively worded items (items 6, 8, 10) and three negatively worded items (items 7, 9, 11) found by Babar and colleagues [21], and the one-factor hypothetical model with 12 items [16]. Moreover, the internal consistency, structural validity, and concurrent validity of the best fit model were also examined.

### 3.1. Participants and Procedure

As Study 1 findings are limited to young adults, Study 2 aimed to address this limitation by recruiting adults in general. A total of 412 adults recruited using convenience sampling responded to the online survey. After removing 54 participants with zero response rate, 358 responses (65.1% females; eight missing values) were submitted for analysis. The participants’ age ranged from 18 to 60 years old (25 missing values) with a mean age of 22.87 (*SD* = 6.07). The sample consisted of 6.9% Malays, 82.5% Chinese, 8.9% Indians, and 1.7% others (e.g., Punjabi, Sino Kadazan). Most of the participants were single (69.8%), followed by the statuses of married (6.6%), and currently in a relationship (23.6%). Seven participants did not report their relationship status. The information sheet and informed consent were presented to the participant before answering the questions. The data collection procedure was reviewed and approved by the Institutional Scientific and Ethical Review Committee.

### 3.2. Measurements

The 12-item FAD-GF and Brief Resilience Scale (BRS) [34] were used in the data collection. The 6-item BRS is a measure of one’s ability to bounce back or recover from stress using a 5-point Likert scale, ranging from 1 (strongly disagree) to 5 (strongly agree). The odd numbers of items were positively worded and even numbers of items were negatively worded. Sample items are “I tend to bounce back quickly after hard times” and “I have a hard time making it through stressful events” (negatively worded). A mean score is computed by averaging the item scores after reverse scoring the negatively worded items. A higher score indicates a higher level of resiliency.

### 3.3. Data Analysis

Confirmatory factor analysis (CFA) with mean- and variance-adjusted weighted least squares (WLSMV) estimator was conducted using the R beta module of the JASP software package version 0.16 [35] to examine the six competing models and identify the best fit model for the FAD-GF. The model fit was assessed using several widely used indices such as ratio of chi-square values to degrees of freedom (χ^2^/df), Comparative Fit Index (CFI), Tucker–Lewis Index (TLI), Root Mean Square Error of Approximation (RMSEA), and standardized root mean square residual (SRMR). A good fit model shall be χ^2^/df < 3, CFI and TLI > 0.95, RMSEA ≤ 0.05, and SRMR < 0.08 [36,37,38]. When a model is barely acceptable, modification indices were consulted for possible improvement.

The internal consistency of the FAD-GF was examined using Cronbach alpha (α) and McDonald’s omega (ω) coefficients. On the other hand, we examined the concurrent validity of the FAD-GF by correlating the FAD-GF score with the BRS score as family functioning has been found to have a positive relationship with resilience (e.g., [5,12,39]).

### 3.4. Results

Ten items (of the FAD-GF) had missing values, hence the pattern of the missing data was investigated. The missing value percentage was lower than 1% for each item. Moreover, the Little’s MCAR test was not significant, χ^2^ (39) = 24.76, *p* = 0.963 thus, missing data were replaced using expectation maximization.

The data were then submitted to CFA to examine and compare the six competing models: three-factor model with 11 items (Model 1), two-factor model with 12 items (Model 2), one-factor model with six negatively worded items (Model 3), one-factor model with six positively worded items (Model 4), two-factor model with 6 items (Model 5), and the hypothetical one-factor model with 12 items (Model 6).

Table 2 summarizes the results of the six models (based on the robust-corrected values). The analysis showed that the indices of Model 1 were acceptable, except for the TLI value: TLI = 0.929. In contrast, Model 2 was found unfit: CFI = 0.901, TLI = 0.877. Both CFI and TLI values were still below the suggested cut-off value even after adding an error covariance between items 3 and 5: CFI = 0.926, TLI = 0.906. A similar pattern was also observed in Model 3: CFI = 0.928, TLI = 0.880. Note that, however, the modified Model 3 with an error covariance between items 3 and 5 (i.e., Model 3a) demonstrated a good fit to the data. Next, Model 4 also showed a good fit, but the TLI value was greater than 1.00: TLI = 1.028. Model 5, on the other hand, showed a good fit to the data. Finally, Model 6 had the worst fit among the six models.

Among the six competing models, three models, Model 3a, Model 4, and Model 5, showed good fit. However, adding an error covariance will limit the usability and interpretation of Model 3a as it is more difficult, especially when structural equation modeling is not used, while the indices of Model 4 imply that the model is overfitting. On the contrary, Model 5 did not require any modification and has a clear-cut result and hence, this two-factor model with 6 items (i.e., FAD-GF-SF) is preferable. The standardized factor loadings of the six items ranged from 0.637 (item 6) to 0.771 (item 7).

To clarify the potential impact of age on the results, based on the existing sample (N = 358), we reran the analysis on the same sample without respondents aged above 29 years old and those who did not report age (N = 309). The results for both samples (N = 358 and N = 309) were consistent, thus the existing sample (N = 358) was used and reported in Study 2.

#### Internal consistency and Structural Validity

The internal consistency of the FAD-GF-SF was assessed using Cronbach’s alpha and McDonald’s omega. Both coefficients of the subscale with negative items were greater than 0.70 (α = 0.752 and ω = 0.756) indicating satisfactory internal consistency, whereas the subscale with positive items had slightly lower coefficients (α = 0.693 and ω = 0.695). On the other hand, the BRS showed poor internal consistency (α = 0.363 and ω = 0.371). Therefore, we did not examine the correlation between FAD-GF-SF and BRS scores.

Taken together, Study 2 shows that the FAD-GF is best represented by the two-factor model with three positively worded and three negatively worded items. Moreover, the FAD-GF-SF showed good internal consistency among adults in Malaysia.

## 4. Discussion

Unlike other measurements of family functioning (e.g., Family Environment Scale, Family APGAR), the FAD-GF has shown inconsistent factor structure. Therefore, the present study investigated the psychometric properties of the English version of the 12-item McMaster Family Assessment Device–General Functioning Subscale (FAD-GF) [16] in two studies using two different groups of Malaysian adults. Our findings did not support the hypothetical structure of the FAD-GF but revealed a new short-form of the FAD-GF consisting of six items (i.e., FAD-GF-SF).

Study 1 explored the factor structure of the FAD-GF using EFA. The EFA results (Study 1) are not aligned with the expected unidimensionality of the 12-item FAD-GF [16]. Instead, we found four alternative models. First, a three-factor solution with 11 items (item 12 was removed due to low factor loading) was found based on the eigenvalue. The negative functioning factor contained four negatively worded items, while the positive functioning factor carried four positively worded items. The last factor, emotional communication, reported three items (items 3, 5, and 6). It is worth noting that Roncone et al. [25] also revealed the emotional communication factor when examining psychometric qualities of the Italian version of the FAD.

Second, the two-factor model recommended by the parallel test consisted of a negative functioning factor with six negatively worded items and a positive functioning factor with six positively worded items. The results are similar to the EFA results of the Malay version of the FAD-GF derived from a sample of Malay parents [21]. However, Babar et al. further validated the scale through CFA and found that the model with three, but not six, items in each factor showed the best fit to their sample [21].

Third, when fixing the number of extractions to one, we found a single factor with six negatively worded items, which has not been reported in the literature. This new solution could be due to our participants being more sensitive to negatively worded items, which is consistent with previous studies that found stronger method effects from the negatively worded items [40,41].

Fourth, we explored the one-factor model with six positively worded items (FAD-GF6+) found by Haan et al. [28]. While a single factor can account for all items, the model showed the lowest total explained variance compared to the three models mentioned above. Taken together, the EFA results imply that the hypothetical unidimensional structure of the FAD-GF may not apply to our sample.

Meanwhile, to clarify the factor structure of the FAD-GF, we employed CFA in Study 2 to examine and compare the six potential models: the above-mentioned four alternative models revealed by EFA, two-factor with six items model [21], and hypothetical one-factor with 12 items model [16]. It was found that (1) the 11-item three-factor model, the 12-item two-factor model, and the hypothetical 12-item one-factor model were unacceptable; (2) the one-factor model with six positively worded items was overfit; and (3) the one-factor model with six negatively worded items was acceptable only after adding an error covariance. As a result, only the two-factor model with six items (i.e., FAD-GF-SF) showed the best fit to the data without any further modification. In line with the findings of Study 1, the findings of Study 2 not only indicate that the hypothetical structure of FAD-GF does not apply to our sample, but also justify the necessity of the present study to assess the psychometric properties of the FAD-GF in the Malaysian context.

It is important to note that the two-factor structure is in line with Babar et al.’s findings [21] of the Malay version of the FAD-GF and is supported by parallel test in Study 1. The consistency suggests that the two factors are meaningful to our sample in capturing the positive and negative aspects of family functioning, respectively. Note that, however, the two factors may merely reflect the methodological differences in (positive versus negative) wording of the items which do not have any practical values. It is also noteworthy that, unlike the FAD-GF-SF, the two-factor model with 12 items was not acceptable. One of the possibilities is that some of the (positively and negatively worded) items are insufficient to capture the concept of family functioning to our sample. Taking everything into account, local researchers are urged to examine the extent to which the items in reflecting Malaysian adults’ connotation of family functioning using a qualitative approach. In addition to replacing and modifying the existing items, researchers may consider adding additional items to capture the concept of family functioning.

Aside from having an excellent model fit, the FAD-GF-SF had shown satisfactory internal consistency. However, the concurrent validity of the FAD-GF-SF with resilience remains open. Furthermore, although it was found that the FAD-GF-SF score positively correlated with the BRS score, the results must be interpreted with caution due to the poor internal consistency of the BRS. Nevertheless, the positive association between family functioning and resilience is consistent with past findings [5,12,39] and the MMFF [20]. The latter highlights the significance of family functioning in shaping individuals’ behaviors, such as returning to normality after experiencing stressful experiences (resilience).

## 5. Implications of the Findings

To our best knowledge, this is the first study to examine the psychometric properties of (the English version of) the FAD-GF among adults in Malaysia. Our findings contribute to the literature by pinpointing the one that works for Malaysian samples from several possible structures of the FAD-GF (i.e., two-factor FAD-GF with six items). Researchers can then apply the FAD-GF-SF with confidence to further investigate the antecedents and impacts of family functioning in the local context. Although the positive and negative factors discovered may not have a practical value, our findings serve as a basis for examining the concept of family functioning qualitatively whether family functioning has positive and negative sides in the Malaysian context. Besides that, by demonstrating that the hypothetical 12-item one-factor model does not apply to the Malaysian context, we urge local researchers to examine the psychometric properties of the measurements developed in foreign cultures before applying them. It is supported by some local studies in which items of some established measurements are not suitable for Malaysian samples (e.g., [42,43]). Finally, along the lines of the past findings of local studies (e.g., [44]), the results of Study 1 and Study 2 suggest that our participants may encounter cognitive challenges in responding to positively and negatively worded items concurrently. Therefore, the findings serve as a reference for local researchers when designing items for a new measurement.

## 6. Limitations and Suggestions for Future Studies

The present study has several limitations. First, the validity of the FAD-GF-SF was not appropriately and comprehensively tested in the study. The present study only examined structural validity and concurrent validity (by correlating the FAD-GF-SF and BRS scores). Although the result is promising, the BRS was found to have poor internal consistency. Future researchers are thus recommended to further examine concurrent validity and predictive validity of the FAD-GF-SF. Second, although the FAD-GF-SF is superior to other competing models, it is important to note that the results were based on the full 12-item version of the FAD-GF. It is, therefore, essential for future researchers to replicate the study using the FAD-GF-SF on a new sample and in different cultural contexts to further shed light on the psychometric properties of the scale. Finally, the sample in Study 1 and Study 2 primarily comprised Chinese young adults and, while Study 2 results suggest that FAD-GF-SF also works for adults, the findings shall be interpreted with caution due to the small sample size. Future researchers are suggested to replicate the study on an adult sample with an equal number of Malay, Chinese, and Indian participants to enhance the generalizability of the results.

## 7. Conclusions

Among the factor structures found in the literature, the two-factor model with six items (i.e., FAD-GF-SF) works best for the Malaysian samples. The preliminary results suggest that the FAD-GF-SF is a reliable and useful tool for assessing family functioning in the Malaysian context.

## Figures and Tables

**Table 1:** Summary of exploratory factor analysis results in Study 1.

Item		M (SD)	Skewness	Kurtosis	Model 1: 3-Factor ^a^				Model 2: 2-Factor ^b^			Model 3: 1-Factor ^c^		Model 4: 1-Factor ^d^	
					1	2	3	h^2^	1	2	h^2^	1	h^2^	1	h^2^
1	Planning family activities is difficult because we misunderstand each other	2.79 (0.87)	−0.32	−0.57	**0.542**	−0.132	0.185	0.389	**0.673**	−0.141	0.405	**0.621**	0.386	-	-
2	In times of crisis we can turn to each other for support	3.31 (0.67)	−0.74	0.67	0.003	**0.404**	0.083	0.194	0.007	**0.470**	0.223	-	-	**0.475**	0.225
3	We cannot talk to each other about the sadness we feel	2.79 (0.88)	−0.34	−0.57	0.159	−0.042	**0.594**	0.447	**0.482**	0.109	0.282	**0.514**	0.265	-	-
4	Individuals are accepted for what they are	3.09 (0.75)	−0.64	0.34	−0.041	**0.639**	0.022	0.408	−0.058	**0.624**	0.367	-	-	**0.607**	0.369
5	We avoid discussing our fears and concerns	2.61 (0.86)	−0.14	−0.62	0.206	−0.107	**0.520**	0.376	**0.494**	0.030	0.256	**0.504**	0.254	-	-
6	We can express feelings to each other	3.03 (0.78)	−0.47	−0.19	−0.195	0.381	**0.543**	0.487	0.091	**0.549**	0.346	-	-	**0.560**	0.314
7	There are lots of bad feelings in the family	3.00 (0.88)	−0.51	−0.56	**0.691**	0.074	0.038	0.535	**0.701**	0.027	0.506	**0.709**	0.503	-	-
8	We feel accepted for what we are	3.12 (0.71)	−0.45	−0.04	0.097	**0.747**	−0.120	0.543	−0.001	**0.649**	0.421	-	-	**0.659**	0.434
9	Making decisions is a problem in our family	2.79 (0.92)	−0.36	−0.68	**0.638**	0.076	0.009	0.441	**0.639**	0.008	0.412	**0.642**	0.413	-	-
10	We are able to make decisions about how to solve problems	3.12 (0.65)	−0.49	0.71	0.128	**0.407**	−0.027	0.196	0.072	**0.417**	0.200	-	-	**0.443**	0.196
11	We do not get along well with each other	3.14 (0.85)	−0.70	−0.27	**0.717**	0.083	−0.029	0.528	**0.685**	−0.001	0.469	**0.688**	0.473	-	-
12	We confide in each other	2.89 (0.74)	−0.35	−0.06	-	-	-	-	−0.077	**0.439**	0.175	-	-	**0.413**	0.170
	Kaiser–Meyer–Olkin				0.804				0.810			0.828		0.747	
	Bartlett’s test				χ^2^ (55) = 1026.02, *p* < 0.001				χ^2^ (66) = 1089.33, *p* < 0.001			χ ^2^ (15) = 601.50, *p* < 0.001		χ^2^ (15) = 376.47, *p* < 0.001	
	Explained total variance (%)				56.62				44.50			48.19		39.96	

Note. N = 417. M = mean, SD = standard deviation, h^2^ = communality. Boldface factor loadings are greater than 0.40. ^a^ Factor 1 = negative functioning, Factor 2 = positive functioning, Factor 3 = emotional communication, ^b^ Factor 1 = negative functioning, Factor 2 = positive functioning, ^c^ Factor 1 = negative functioning, ^d^ Factor 1 = positive functioning.

**Table 2:** The fit indices for the alternative models of the McMaster Family Assessment Device-General Functioning Subscale using WLSMV.

Model		χ^2^	df	*p*	χ^2^/df	CFI	TLI	RMSEA [90% CI]	SRMR
1	3-factor with 11 items ^a^	62.786	38	0.007	1.65	0.951	0.929	0.043 [0.022–0.061]	0.048
2	2-factor with 12 items	104.307	53	<0.001	1.97	0.901	0.877	0.052 [0.037–0.067]	0.059
2a	2-factor with 12 items (error covariance between items 3 and 5)	90.284	52	0.001	1.74	0.926	0.906	0.045 [0.029–0.061]	0.055
3	1-factor with 6 negatively worded items	39.614	9	<0.001	4.40	0.928	0.880	0.098 [0.068–0.130]	0.050
3a	1-factor with 6 negatively worded items (error covariance between items 3 and 5)	9.133	8	0.331	1.14	0.997	0.995	0.020 [0.000–0.067]	0.023
4	1-factor with 6 positively worded items	6.030	9	0.737	0.67	1.000	1.028	0.000 [0.000–0.043]	0.024
5	2-factor with 6 items [21]	11.069	8	0.198	1.38	0.985	0.973	0.033 [0.000–0.075]	0.033
6	1-factor with 12 items [16]	247.945	54	<0.001	4.59	0.626	0.543	0.100 [0.088–0.113]	0.148

Note. N = 358. CFI = comparative fit index, TLI = Tucker–Lewis index, RMSEA = root mean square error of approximation, CI = confidence interval, SRMR = scaled standardized root mean residual. ^a^ the covariance matrix of the residuals of the observed variables (theta) is not positive definite.

## Data Availability

The data that support the findings of this study are available from the corresponding author, upon reasonable request.
